# Measuring unconscious cognition: Beyond the zero-awareness criterion

**DOI:** 10.2478/v10053-008-0030-3

**Published:** 2008-07-15

**Authors:** Thomas Schmidt

**Affiliations:** Department of Cognitive Psychology, University of Giessen, Germany

**Keywords:** response, priming, masking, visual awareness, dissociations, feedforward sweep

## Abstract

Visual masking can be employed to manipulate observers’ awareness of critical stimuli in studies of masked priming. This paper discusses two different lines of attack for establishing unconscious cognition in such experiments. Firstly, *simple dissociations* between *direct measures (D)* of visual awareness and *indirect measures (I)* of processing per se occur when *I* has some nonzero value while *D* is at chance level; the traditional requirement of zero awareness is necessary for this criterion only. In contrast, *double dissociations* occur when some experimental manipulation has opposite effects on *I* and *D*, for instance, increasing priming effects despite decreasing prime identification performance (Schmidt & Vorberg, 2006). Double dissociations require much weaker measurement assumptions than other criteria. An attractive alternative to this dissociation approach would be to use tasks that are known to violate *necessary conditions* of visual awareness altogether. In particular, it is argued here that priming effects in speeded pointing movements (Schmidt, Niehaus, & Nagel, 2006) occur in the absence of the recurrent processing that is often assumed to be a necessary condition for awareness (for instance, DiLollo, Enns, & Rensink, 2000; Lamme & Roelfsema, 2000). Feedforward tasks such as this might thus be used to measure the time-course of unconscious processing directly, before intracortical feedback and awareness come into play.

##  

Attempts to demonstrate unconscious processing are as old as experimental psychology itself (e.g., Peirce & Jastrow, 1884). Given this long history, it is puzzling that the topic appears just as controversial today as it did decades ago (Erdelyi, 2004; Eriksen, 1960; Holender, 1986; Holender & Duscherer, 2004). Paradoxically, this controversy does not so much concern the *existence* of unconscious processing (most researchers seem to be convinced of this) but the question how to *demonstrate* unconscious processing in a given experiment.

Progress in the field has been handicapped by the unquestioned assumption that in order to demonstrate unconscious processing, one has to make sure that a critical stimulus was completely outside of awareness. In this contribution, *I* would like to propose two alternative lines of attack for establishing unconscious processing beyond the zero-awareness criterion. The first part of the paper will deal with different types of dissociation between measures of awareness and measures of processing *per se* (Schmidt & Vorberg, 2006). The conclusion of this section is that even though different methods are available, the most powerful approach involves *double dissociations* where an experimental manipulation is shown to have opposite effects on the two measures. Surprisingly, it can be shown that this type of dissociation does not require, nor does it benefit from, unconscious stimuli. In the second part of the paper, I will focus on the possibility of working out the *necessary conditions* for awareness: If these conditions be known, measures known to defy them could be used to measure unconscious processing directly. As an illustration, I will argue that priming effects in speeded pointing movements (Schmidt, Niehaus, & Nagel, 2006) occur in the absence of the recurrent processing that is often assumed to be a necessary condition for awareness (for instance, DiLollo, Enns, & Rensink, 2000; Lamme, 2002; Lamme & Roelfsema, 2000; Tong, 2003).

## Simple dissociations and the zero-awareness criterion

To demonstrate that a critical stimulus was processed unconsciously, one usually has to produce some dissociation between different behavioral measures of performance concerning that stimulus. Traditionally, this is done by comparing two measures obtained from different tasks.^1^ One measure (called the *direct measure, D*) is supposed to signal the observer’s awareness of the critical stimulus, for instance, in a forced-choice prime discrimination task. The second measure (called *indirect measure, I*) is used as an indicator that the stimulus was processed at all, for instance, a priming effect in reaction times. The traditional criterion for unconscious processing has required *D* to equal zero, assuming that this signals the absence of any conscious processing of the critical stimulus. At the same time, *I* is required to be nonzero, indicating that the stimulus was nevertheless processed (Reingold & Merikle, 1988; Shanks & St. John, 1994). Historically, this zero-awareness criterion has run into difficulties because it only works if a valid conclusion can be drawn from *zero performance in the direct measure* to *zero awareness in the observer* (Reingold & Merikle, 1988, 1990, 1993; Reingold, 2004).

Recently, Dirk Vorberg and *I* have examined the scopes and assumptions required by the zero-awareness criterion as well as alternative approaches (Schmidt & Vorberg, 2006). We start from the assumption that direct as well as indirect measures may depend on two sources of stimulus information which may be labeled “conscious” *(c)* and “unconscious” *(u)* without loss of generality: *D = D(c, u)*, *I = I(c, u)*, where information is defined non-negative. The dependency is supposed to be weakly monotonic, which means that if any type of information increases, the measures can only increase or remain constant (in the long run, that is, in the expected values). These are weak assumptions that must be conceded for virtually any measurement situation. Establishing unconscious processing then consists in refuting a Null Model which states that the influence of unconscious information is zero, or equivalently, that both measures are driven by a single source of conscious information. If the null model is discarded, performance in the two tasks must be driven by at least one additional source of information.

There is one important constraint here. If *D* and *I* are to be modeled as functions of the same arguments *c* and *u*, one has to make sure that the underlying conscious and unconscious information is the same for both measures. Therefore, the direct and indirect tasks must be designed to use identical stimuli, identical responses, and identical stimulus-response mappings (Schmidt & Vorberg, 2006). In other words, *D* must address *exactly that stimulus distinction that drives the effect in the indirect task* (Reingold & Merikle, 1988). For an example of mismatch between direct and indirect tasks, consider the study by Dehaene et al. (1998). The indirect task was to indicate as quickly as possible whether a target digit was numerically smaller or larger than five, where the target digit was preceded by a masked prime digit. Response times in this task were shorter when the prime was consistent with the target (i.e., both numbers < 5) than when the prime was inconsistent (i.e., prime < 5 but target > 5). The optimal direct task would have asked for the same feature discrimination, namely deciding whether the prime was larger or smaller than five, because this was the information driving the priming effect. Instead, the authors employed *two* direct tasks, detection of the primes against an empty background, and discrimination of the primes from random letter strings, none of which captured the critical distinction of whether the prime was smaller or larger than five. A more subtle example is from the seminal study by Neumann and Klotz (1994). In the indirect task, participants performed a speeded discrimination of whether a square was presented to the left or right of a diamond, so that each of the two stimulus alternatives was mapped onto exactly one response. This target pair was preceded by a smaller pair of diamond and square in either the same (consistent) or the reverse (inconsistent) configuration, or by a neutral prime pair (e.g., two diamonds). In the direct task, participants had to classify the prime pairs as neutral vs. non-neutral, such that the neutral prime pair was now mapped onto one response, and *both* remaining prime pairs onto the other response. Even though the direct and indirect tasks employed identical stimuli, the direct task used a more complex and presumably more difficult stimulus-response mapping.

**Figure 1.**
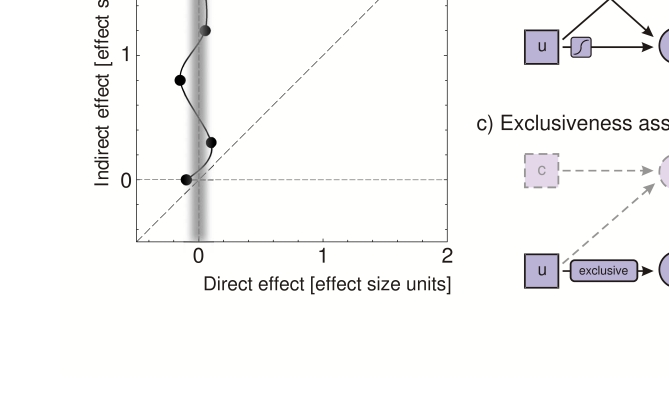
Data patterns and assumptions necessary to interpret a simple dissociation as evidence for nonzero unconscious information. An arrow from information source *a* to measure *B* indicates that *B* is some function of *a*. *S*-shaped inset symbols denote that weak monotonicity is assumed for that function. Abbreviations as explained in main text. **a**) Data pattern required for a simple dissociation. Direct and indirect measures are plotted in an opposition space in effect size units. Evidence for a simple dissociation is given by data points lying on the stippled vertical line such that *I* > 0 while *D* = 0. **b**) A simple dissociation gives evidence for nonzero unconscious information if it can be assumed that *D* is an exhaustive function of *c* and that *I* is a weakly monotonic function of *u*. **c**) Alternatively, a simple dissociation gives evidence for nonzero unconscious information if *I* is an exclusive measure of *u*. Adapted from Schmidt and Vorberg (2006).

Given that *D-I* mismatch is efficiently avoided, how can the null model of only conscious processing be disproved? The traditional way of doing this is the zero-awareness criterion, which produces what we call a *simple dissociation* of direct and indirect measures: zero *D* in the presence of nonzero *I* (Figure 1). If we start from this finding, we quickly see that we don’t get very far without additional assumptions, because the observation that *I(c, u)* > 0 only implies that *c* > 0, *u* > 0, or both. Can we use the fact that *D(c, u)* = 0 to make sure that *c* = 0? Not quite, because *D(c, u)* = 0 does not imply *c* = 0 under weak monotonicity assumptions: *D* may simply fail to respond to changes in information, so that there could be some *c* that *D* was not able to detect. To work around this problem, we have to make the stronger assumption that *D* is an *exhaustive* measure of conscious information, that is, that *D* is a *strictly monotonic* function of *c* (Reingold & Merikle, 1988; see Schmidt & Vorberg, 2006, for a more general proof). This means that *D* is able to detect any change in *c* whatsoever, so that *D(c, u)* = 0 implies *c* = 0. Given this *exhaustiveness assumption*, we can finally use the fact that *c* can no longer drive the indirect effect: *I(c, u)* = *I(0, u)* > 0 implies *u* > 0, which says that there is nonzero unconscious information in the system.

How restrictive is the exhaustiveness assumption? It requires that no change in awareness, however small, must escape detection by *D*; only then can we infer the absence of awareness from zero values in the direct measure. You may compare this with your old mechanical barometer which is likely to be a weakly monotonic measure of atmospheric air pressure: The needle of the barometer tends to rise with air pressure, but it sometimes “hangs”, and you have to knock against the shell to break the needle free. A strictly monotonic, exhaustive measure of air pressure would be an infinitely sensitive barometer, one that never hangs. Strict monotonicity is violated by conditions as trivial and inescapable as random error in the direct measure. The exhaustiveness assumption is thus a strong requirement that should not be taken for granted. If the exhaustiveness assumption is wrong, it can always be argued that it was conscious processing alone that influenced both *D* and *I*, but that *I* was sensitive enough to detect it while *D* was not (Reingold & Merikle, 1988).

There are some other difficulties with simple dissociations that are more on the practical side. One often stated problem is how to show statistically that *D* is not different from zero, because this involves “proving the null hypothesis”, which is a commonplace problem in scientific research. Actually, the solution to this is straightforward and requires establishing binding criteria for effect, size, power, or confidence limits in the direct measure (Murphy & Myors, 1998).^2^ However, given the conservativeness of applied statistics, this is unlikely to happen soon. Another practical problem is that finding stimulus conditions that will yield chance performance in the direct task is difficult, and largely a matter of good luck.

There is an alternative set of assumptions that abolishes the need for a direct measure altogether (Fig. 1c). This is when the indirect measure can be assumed to be an *exclusive measure* of unconscious information, that is, a weakly monotonic function of *u* that is unaffected by *c*. In this case *I(c, u)* = *I(u)* > 0 implies *u* > 0 directly.^3^ Tentative evidence for exclusive measures of unconscious processing is discussed later in this paper.

## Beyond zero awareness I: Double dissociations

**Figure 2.**
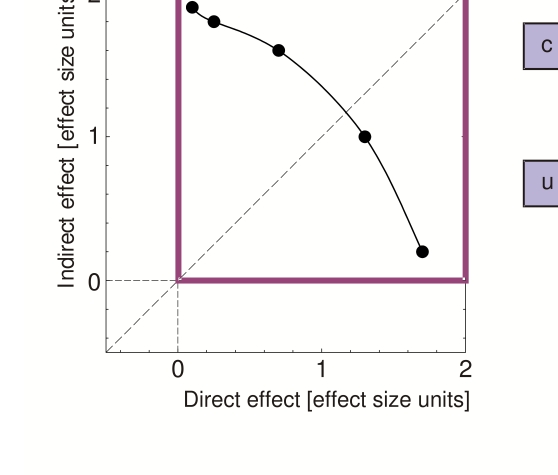
Data patterns and assumptions necessary to interpret a double dissociation as evidence for nonzero unconscious information. **a**) Data pattern required for a double dissociation. Evidence for a double dissociation is given by any pair of data points that can be connected by a straight line with negative slope anywhere in *D*-*I* space. **b**) A double dissociation gives evidence for nonzero unconscious information if it can be assumed that *I* and *D* are weakly monotonic functions of *c*. Further assumptions need not be made, leaving *c* and *u* to interact freely on both measures. Adapted from Schmidt and Vorberg (2006).

One interesting way to circumvent the exhaustiveness or exclusiveness assumptions is to let awareness vary over experimental conditions. It may then be possible to establish a *double dissociation*, which consists of finding an experimental manipulation that changes *D* and *I* in opposite directions (Figure 2). In particular, any pair of experimental conditions that leads to opposite orderings of data points in direct and indirect measures gives evidence for a double dissociation. An example would be a priming experiment with two (or more) masking conditions where the priming effect *increases* over experimental conditions while prime identification performance *decreases*. It is intuitively clear that two measures of visual information going in opposite directions cannot be monotonically driven by a single information source, and a formal proof of this can be found in our paper (Schmidt & Vorberg, 2006)^4^. Our concept of double dissociations is analogous to the widely used methodology in neuropsychology and medicine (Shallice, 1988; Sternberg, 2001).

Double dissociations have surprising features (see Schmidt & Vorberg, 2006, for details). Firstly, they require *D* to be nonconstant: They cannot be obtained in the complete absence of awareness but require variation of awareness over a range of experimental conditions, so that there must be nonzero awareness for the prime under at least some conditions. Secondly, double dissociations require weaker assumptions than simple dissociations: There is no need for an exhaustiveness or an exclusiveness assumption, and we can even drop the assumption of weak monotonicity for all functions of *u*. Adopting the barometer metaphor from the last section, not only is the direct measure allowed to “hang” with respect to conscious information, but neither direct nor indirect measures have to be monotonically related to unconscious information at all. Because of this, *c* and *u* are allowed to produce arbitrary interactive effects on *D* and *I* like, for instance, when *c* and *u* are mutually inhibitory (Snodgrass, Bernat, & Shevrin, 2004; see Schmidt & Vorberg, 2006, for proof). The surprising outcome is thus that unconscious stimuli are not required for demonstrating unconscious processing.

Examples of simple as well as double dissociations come from experiments in *response priming* (Neumann & Klotz, 1994; see also Ansorge & Neumann, 2005; Dehaene et al., 1998; Eimer & Schlaghecken, 1998, 2003; Jaśkowski, van der Lubbe, Schlotterbeck, & Verleger, 2002; Klotz & Neumann, 1999; Leuthold & Kopp, 1998; Mattler, 2003; Schmidt, 2002; Verleger, Jaśkowski, Aydemir, van der Lubbe, & Groen, 2004). In experiments by Vorberg, Mattler, Heinecke, Schmidt, and Schwarzbach (2003, 2004), participants performed speeded keypress responses to the direction of an arrow-shaped masking stimulus that was preceded by an arrow-shaped prime. The mask had a dual purpose here, acting as the target of the response and at the same time reducing visibility of the prime by metacontrast, a form of visual backward masking (Breitmeyer & Öğmen, 2006; Francis, 1997). As the stimulus-onset asynchrony (*SOA*) between prime and mask increased, priming effects also increased, such that primes pointing into the same direction as the mask shortened response times, while primes pointing into the opposite direction prolonged them. Strikingly, this priming effect was independent of visual awareness of the prime. We determined this by using stimulus conditions that produced different time-courses of metacontrast masking. When a 17-ms prime was followed by a 140-ms mask, primes were virtually invisible, and participants were unable to perform better than chance when asked to discriminate the pointing direction of the prime (in over 3,000 trials per participant). These findings provide strong evidence for a simple dissociation as traditionally required. In a second experiment, however, we compared all four pairings of short-duration (14 ms) and long-duration (42 ms) primes and masks, yielding very different types of masking functions. When 14-ms primes were combined with 42-ms masks, prime identification performance was low and slightly increased with SOA; performance was better when mask duration was reduced to 14 ms. When a 42-ms prime was paired with a 14-ms mask, prime identification performance was nearly perfect. However, a 42-ms prime combined with a 14-ms mask yielded an effect called *type-B masking* (Breitmeyer & Öğmen, 2006), where prime identification performance markedly *decreases* with the prime-mask SOA, then increases again. Still, the priming effect increased monotonically, producing a strong double dissociation between priming and prime identification performance. These data defy the claim that direct and indirect measures tend to convey similar amounts of information about the critical stimulus (Franz, 2006): Priming increased linearly with SOA no matter whether the prime was completely visible, completely invisible, or whether visibility increased or decreased with SOA. Clearly, this data pattern reveals a relationship that would never have been found by simple dissociation: Response priming is *independent* of prime identification performance, with different time-courses in the two tasks.

There are further examples of double dissociations in masked priming studies. Mattler (2003) reports a series of experiments where not only motor responses were primed but also shifts in visual attention and task set. Double dissociations were evident in the time-course of linearly increasing priming effects under type-B masking conditions. Further examples of double dissociations include Merikle and Joordens’ (1997a, b) demonstration of *qualitative dissociations* (Merikle & Cheesman, 1987). These authors used a variant of the Stroop (1935) task where participants responded to the color of red or green target stimuli (strings of ampersands) that were preceded by the prime words “RED” or “GREEN”. The regular Stroop effect features faster responses in consistent trials (e.g., “RED - &&&&&&&”) than in inconsistent trials (e.g., “RED - &&&&&&&”). However, when most of the primes are inconsistent with the target, participants often use the resulting contingency and eventually respond faster in inconsistent than in consistent trials. However, the authors found this reversal only under conditions of weak visual masking: When the primes were strongly masked, only the regular effect was observed. Such “qualitative dissociations” can be interpreted as special cases of double dissociations (Schmidt & Vorberg, 2006, mathematical appendix).

Searching for double dissociations has practical implications. It requires setting up different conditions of prime visibility, thereby encouraging the employment of parametric experiments. In particular, it is often advantageous to study the full time-course of priming and masking over the SOA range of interest, because sampling the priming process at only one point in time may lead to grossly misleading conclusions if the time-course changes across experimental conditions (Lingnau & Vorberg, 2005). A similar point can be made for simple dissociations: Demonstrating that the direct measure is invariant over a range of conditions despite marked changes in the indirect measure is often more convincing than looking at only one experimental condition and argue that *D* has a specific value, zero. Thus, even in cases where double dissociations are hard to find, parametric experimentation can do a lot to improve the cogency of the data. Visual masking procedures that lead to decreases in visibility with increasing prime-mask SOA (for example, DiLollo et al., 2000; Francis, 1997; Francis & Herzog, 2004) are of special interest for the establishment of double dissociations.^5^

## Beyond zero awareness II: Violating necessary conditions for awareness

Dissociations at the task level are able to provide only indirect evidence for a distinction of underlying processes. An exciting alternative would be to work out the necessary conditions for visual awareness and then try to find behavioral tasks that violate those conditions. In other words, we could search for indirect measures that are exclusive measures of unconscious processing.

In a metaanalysis of 48 studies investigating the response latencies of various cortical areas to a sudden visual stimulus, Lamme and Roelfsema (2000) showed that the stimulus creates a wave of activation traveling from posterior to anterior areas, reaching most cortical areas within about 150 ms, including prefrontal and primary motor cortices. The authors estimated that this leaves cells with only about 10 milliseconds’ time to pass their own activation on to later areas, which is about the duration of a typical interspike interval. Therefore, if most cells have to pass on their activation with the next spike fired, there is little or no time to integrate feedback from other cells. Based on this, Lamme and Roelfsema suggested that the first wave of visual activation travels through the system as a *fast feedforward sweep* whose wavefront is essentially free of intracortical feedback information. This is well in line with behavioral measurements from rapid stimulus classification tasks as well as neural network simulations, which suggest that most of the stimulus-relevant information could be extracted from the temporal distribution of the very first spikes in the feedforward wavefront, (Kirchner & Thorpe, 2006; Rousselet, Fabre-Thorpe, & Thorpe, 2002; VanRullen & Koch, 2003; VanRullen & Thorpe, 2002).^6^

Lamme and Roelfsema (2000; Lamme, 2002) assume that feedforward processing alone is not sufficient for generating visual awareness. Along with several other authors (for instance, DiLollo et al., 2000; Tong, 2003), they propose that conscious perception is possible only with recurrent processing of the stimulus. Evidence for this view comes from studies indicating that visual awareness of a stimulus is suppressed if feedback loops from extrastriate visual areas through primary visual cortex are disrupted at critical points in time, for instance, by a visual backward mask (Bacon-Macé, Macé, Fabre-Thorpe, & Thorpe, 2005; Lamme, Zipser, & Spekreijse, 2002; Macknik & Haglund, 1999; Macknik & Livingstone, 1998) or by transcranial magnetic stimulation (Pascual-Leone & Walsh, 2001; Ro, Breitmeyer, Burton, Singhal, & Lane, 2003). This view would be able to explain the major findings in response priming: Priming could reflect visuomotor activation transmitted by the fast feedforward sweeps initiated by primes and masks before recurrent processes set in to gradually wipe out the prime signal before it enters visual awareness. The feedforward processes associated with priming should therefore be independent of the recurrent processes leading to visual awareness and backward masking, which is nicely compatible with the double-dissociation findings by Vorberg et al. (2003, 2004) and Mattler (2003) as well as the abundant evidence from simple dissociations.

**Figure 3.**
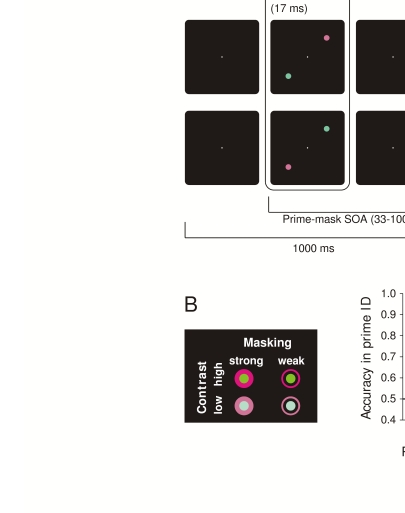
**a**) Experimental tasks and procedures employed by Schmidt (2002) and Schmidt et al. (2006). **b**) Stimulus conditions were varied by crossing two levels of color contrast (for primes and masks together) with two types of masks (“strong” metacontrast masks or “weak” pseudomasks). **c**) Stimulus conditions had large effects on prime identification performance. The 17-ms SOA condition refers to a second experiment not reported here. Adapted from Schmidt et al. (2006).

To convincingly link response priming to feedforward processing, one has to show that visuomotor activation is not only transmitted rapidly, but that the dynamics of this transmission are consistent with a feedforward process. Evidence for rapid visuomotor transmission in masked priming comes from the study of primed pointing responses (Schmidt, 2002; see also Brenner & Smeets, 2004). In that study, participants were presented with one red and one green prime flashed simultaneously in opposite quadrants of the display, followed by one red and one green metacontrast mask (annuli that closely fitted around the primes) at the same locations as the primes (Figure 3a). Primes and masks at corresponding positions could either have the same colors (consistent primes), or prime colors could be switched compared to mask colors (inconsistent primes). Participants had to point as quickly as possible from the fixation point towards the mask with appointed color (*Mask ID* task, designed to measure response priming effects), or tried to point without time pressure to the position where they believed the prime of that color had occurred (*Prime ID* task, designed to assess visual awareness of the primes).

Results clearly showed that responses were controlled consecutively by prime and mask signals even when the primes were completely masked. Pointing responses started at a fixed time following prime onset and initially went into the direction specified by the primes, even though the mask signals were the actual targets of the response. When primes and masks were consistent, this initial direction was correct, and the finger simply travelled towards the correct mask until the response was completed. When primes and masks were inconsistent, however, the finger initially traveled into the quadrant occupied by the misleading prime. This detour into the wrong quadrant lasted for a time depending on prime-mask SOA; then the finger stopped and finally proceeded in the correct direction. These data suggest that pointing movements are under continuous control of the color stimuli responded to: Prime signals reach motor areas of the brain in advance of the mask signals, governing the initial phase of the pointing response, whereas mask signals are able to take control in mid-flight with a delay depending on the prime-mask SOA. Moreover, these signals seem to travel fast enough to escape visual masking processes, because priming effects occurred even when prime ID performance was at chance.

So far, these results only tell us that response control in primed pointing movements occurs very rapidly, but is it purely feedforward? If the notion of a feedforward sweep is applied strictly, each cell first reached by the feedforward wavefront can respond to it only on the basis of its preestablished input-output properties (its classical receptive field). The feedforward hypothesis in this strong form is controversial, because feedback mechanisms in early visual areas can be very rapid (Bullier, 2001; Girard, Hupé, & Bullier, 2001), and there are many possibilities for signals processed in parallel visual streams to cross or overtake each other well before the first overt signs of motor activation (Merigan & Maunsell, 1993). It is therefore worthwhile to step back a bit and focus at the input-output dynamics of the system as a whole instead of claiming purely feedforward processing in all its subcomponents. To do this, we introduced the concept of a *rapid chase* (Schmidt et al., 2006). This concept applies to visuomotor tasks where sequential visual stimuli run for control of the same speeded motor response – for instance, when a pointing response is initiated by one stimulus and then altered in mid-flight by an immediately following stimulus (Brenner & Smeets, 2004; Schmidt, 2002). By our definition, two successive visuomotor signals are said to be engaged in a rapid chase if

*(1)* the response is initiated by the first stimulus,

*(2)* the response is influenced by the second stimulus before it is completed, and

*(3)* the response to the first stimulus is initially independent of the second stimulus.

These *rapid-chase criteria* say that if successive signals are in a rapid chase, they will take strictly successive control over the same motor response, such that the response will initially be controlled by the first stimulus alone. The third criterion is crucial because it demands sequential stimulus signals to exert strictly sequential response control.

We adopted the pointing task used by Schmidt (2002) , employing two different types of masking stimuli, which could either be efficient annular metacontrast masks fitting snugly around the prime, or thin annular pseudomasks that left a large gap around the outer contours of the prime (Figure 3b). We also varied the overall color contrast of all stimuli (primes and masks together). Results replicated all the basic findings reported earlier (Schmidt, 2002) and met all three of the rapid-chase criteria. Firstly, responses to the mask stimuli were actually triggered by the prime, as shown by the fact that pointing onset was time-locked to prime rather than mask onset and that the finger tended to detour into the quadrant occupied by the misleading prime. Secondly, mask stimuli took over the response in midflight, so that even responses detouring into the wrong direction were captured after a time depending on the prime-mask SOA and redirected into the correct direction.

**Figure 4.**
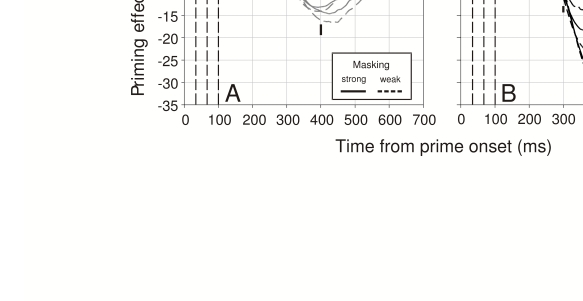
Priming effects in pointing movements in the mask identification task of Schmidt et al. (2006). **a**) Low-contrast color conditions. **b**) High-contrast color conditions. Note that the time axis is locked to prime onset while the possible times of mask onsets are indicated by the stippled vertical lines. Priming effects were calculated by subtracting finger positions in consistent and inconsistent trials. Vertical bars correspond to within-subject standard errors at several points in time, pooled across subjects. Note that in both color contrast conditions, the initial time-course of priming is identical for all SOAs and both mask types, strongly suggesting that early priming effects exclusively depend on properties of the prime but are independent of all mask characteristics. Adapted from Schmidt et al. (2006).

To assess the validity of the crucial third criterion (the response’s initial independence of the mask stimulus), we derived a spatial measure of the priming effect by subtracting the finger positions in consistent and inconsistent trials. (This measure tells us how far the finger position in inconsistent trials lags behind the finger position in consistent trials at corresponding points in time.) For both high-contrast and low-contrast color stimuli, spatial priming effects started to develop at a time locked to prime onset, and priming effects became larger for longer SOAs and weaker masks (Figure 4). Strikingly, however, all these priming functions were initially the same, neatly conforming to our third rapid-chase criterion: The early time-courses of priming were virtually identical for all combinations of mask type and SOA, exclusively depending on characteristics of the prime but being completely independent of all mask characteristics.^7^

To see the significance of this invariance, which was obvious in each participant, consider a general model of priming where the pointing movement at the onset of the priming effect is controlled by information coming from the mask as well as the prime, indicating an early mixture of signals. Under such a model, the initial slopes of the priming trajectories should be smaller for shorter prime-mask SOAs and for stronger masks, because these factors should increase the influence of the mask signal relative to the prime signal and thus reduce the priming effect. In other words, the presence of recurrent information in the earliest parts of the motor signal would be expected to contain information from the mask and therefore to dampen the early time-course of the priming effect. In contrast, the finding that this time-course is initially invariant indicates that the mask signal has no influence whatsoever at the time when the prime first affects the pointing movement. This finding strongly suggests that early priming effects are based on signals carrying only prime but no mask information.

Data from primed pointing movements thus meet all our requirements for a rapid chase: Sequentially presented visual signals control pointing movements in a strictly sequential fashion, and the prime- and mask-triggered visuomotor signals never seem to mix. This finding provides independent behavioral evidence for an early phase of visuomotor processing that is at least primarily if not entirely feedforward (Lamme & Roelfsema, 2000)^8^. At the same time, it establishes response priming of pointing movements (and presumably a much larger class of speeded visuomotor tasks) as a candidate for a feedforward task that proceeds in the absence of recurrent processing. If recurrent activity really turns out to be a necessary condition for visual awareness, such feedforward tasks should be devoid of conscious information, that is, be exclusive measures of unconscious processing.

## Where do we go from here?

In this paper I have argued for two very different strategies to circumvent the difficulties associated with the zero-awareness criterion. One way is to find dissociation patterns that go beyond that criterion. If a double dissociation between direct and indirect measures can be established, this provides an even stronger argument for unconscious processing than does the traditional simple dissociation, because the double dissociation approach rests on much milder assumptions (Schmidt & Vorberg, 2006). An exciting and increasingly viable alternative is to use accumulating evidence from behavioral neuroscience about the necessary conditions for visual awareness, for instance, the requirement for recurrent processing. Behavioral measures could then be developed that are known to violate these conditions, for instance, by meeting the rapid-chase criteria (Schmidt et al., 2006). This approach does not hinge on finding tricky dissociation patterns or by leaning heavily on measurement-theoretical assumptions, but on gradually converging evidence from neuroanatomy, physiology, psychophysics, and behavioral measurement.

It is still unclear how far the recurrent-processing hypothesis will actually carry, and for the time being, the dissociation approach is probably still the safer bet. But measurement theory can only take us so far. In order to use dissociations for demonstrating unconscious processing, one has to work from the assumption that the “conscious/unconscious” distinction is valid in the first place. All that dissociations can teach us is that a single source of information is not sufficient to explain the data, including a single source of “conscious” information. But the insight that there must be at least two sources does not by itself imply that one of them is unconscious: There might be two dissociable types of conscious (or unconscious) information. One reviewer of this article asked whether this wouldn’t render the search for double dissociations a fruitless exercise - if true, of course, this conclusion would hold for simple as well as double dissociations. However, the validity of the “conscious/unconscious” distinction is a conceptual issue that is simply beyond the scope of the measurement-theoretical arguments presented by Schmidt and Vorberg (2006) . Whether or not the concept of unconscious processing will stand the test of time or be replaced by a different concept must be the outcome, not the starting point, of scientific investigation. Dissociations at the measurement level provide tools for performing this investigation, and our analysis only shows which of these tools will work best in the widest range of situations.

## Notes

1 For formal proofs and definitions, please refer to Schmidt and Vorberg (2006), especially the mathematical appendix.

2 The regression technique advocated by Greenwald and coworkers (Draine & Greenwald, 1998; Greenwald, Klinger, & Schuh, 1995) is an alternative method for demonstrating simple dissociations that has been strongly criticized on methodological and conceptual grounds (Dosher, 1998; Merikle & Reingold, 1998; Miller, 2000; Schmidt & Vorberg, 2006) and requires all the assumptions of the conventional simple-dissociation logic in addition to the statistical ones introduced by the regression methodology. Arguably, then, this method is worse than the original approach.

3 This exclusiveness assumption must not be confused with the one stated by Reingold and Merikle (1988) . These authors propose that simple dissociations can be interpreted as evidence for unconscious processing only if the direct measure is both exhaustive and exclusive for conscious information. The latter requirement would be highly problematic because unconscious processing is probably a ubiquitous precursor to conscious processing. Fortunately, from our analysis, it is sufficient that *D* be exhaustive for *c* or that *I* be exclusive for *u* – and note that these are alternative sets of assumptions that do not have to be met at the same time. Also note that the proofs given in our paper (Schmidt & Vorberg, 2006) are more general than those in Reingold and Merikle (1988) because they do not assume additivity of conscious and unconscious effects.

4 In our paper, we discuss another type of dissociation, the sensitivity dissociation proposed by Reingold and Merikle (1988) , which only requires the indirect measure to produce larger effects than the direct measure (Schmidt & Vorberg, 2006). The assumptions under which this works are somewhat special, and this dissociation is omitted here for brevity.

5 Historically, most studies have varied the physical energy of the prime (for instance, its contrast, intensity, or duration) to control its visibility. Such manipulations should be avoided because they affect priming and visual awareness of the prime in similar ways. In particular, any disappearance of priming effects as prime energy is reduced does not constitute logically valid evidence against unconscious processing, because it cuts the necessary input to conscious and unconscious processes alike.

6 Note that cells left in the immediate wake of the feedforward sweep may pick up feedback very rapidly. The claim here is not that the entire system is feedback-free for the duration of the feedforward sweep, but that the wavefront of the sweep travels just ahead of rapidly developing recurrent processes.

7 Reanalysis of the data reported in Schmidt (2002) confirmed these results, showing that the findings by Schmidt et al. (2006) are not acciden-tal.

8 The notion of a rapid chase is milder than that of a feedforward sweep: Whereas the feedforward sweep entails the possibility of rapid chases, the rapid chase account allows for local recurrent activity as long as sequential signals still lead to strictly sequential motor output. Therefore, rapid chases suggest but do not logically imply feedforward processing.
